# Evolutionary Optimisation of Beer Organoleptic Properties: A Simulation Framework

**DOI:** 10.3390/foods11030351

**Published:** 2022-01-26

**Authors:** Mohammad Majid al-Rifaie, Marc Cavazza

**Affiliations:** 1 School of Computing & Mathematical Sciences, University of Greenwich, London SE10 9LS, UK; 2 National Institute of Informatics, Tokyo 101-8430, Japan; cavazzam@acm.org

**Keywords:** food personalisation, beer optimisation, recipe discovery, dispersive flies optimisation

## Abstract

Modern computational techniques offer new perspectives for the personalisation of food properties through the optimisation of their production process. This paper addresses the personalisation of beer properties in the specific case of craft beers where the production process is more flexible. Furthermore, this work presents a *solution discovery method* that could be suitable for more complex, industrial setups. An evolutionary computation technique was used to map brewers’ desired organoleptic properties to their constrained ingredients to design novel recipes tailored for specific brews. While there exist several mathematical tools, using the original mathematical and chemistry formulas, or machine learning models that deal with the process of determining beer properties based on the predetermined quantities of ingredients, this work investigates an *automated quantitative ingredient-selection* approach. The process, which was applied to this problem for the first time, was investigated in a number of simulations by “cloning” several commercial brands with diverse properties. Additional experiments were conducted, demonstrating the system’s ability to deal with on-the-fly changes to users’ preferences during the optimisation process. The results of the experiments pave the way for the discovery of new recipes under varying preferences, therefore facilitating the personalisation and alternative high-fidelity reproduction of existing and new products.

## 1. Introduction

While there is much interest in the optimisation of food production processes, the potential of optimisation techniques to generate recipe diversity through multiple solutions has not been fully explored. Taking the brewing process as a test case, we wish to investigate how optimisation techniques can target specific organoleptic properties in a way which also supports the production of novel variants, with the potential of extending product range on a principled basis. The complexity of the brewing process often necessitates a strict adherence to existing recipes and the associated instructions with the aim of reducing incidents and avoiding costly guessworks [1]; this is especially the case when the primary goal is the production of a beer with specific and predetermined organoleptic characteristics.

**Figure 1:**
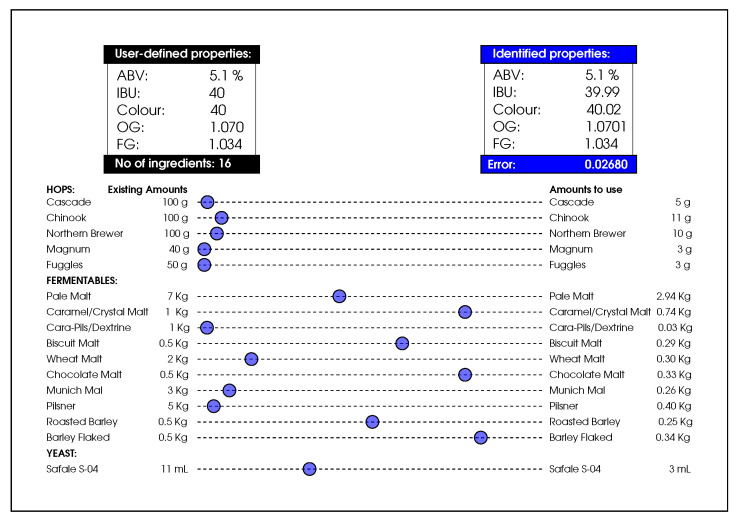
Brewing process optimisation: a schematic view. Users’ desired values are provided to the system using the control panel on the top-left corner. The corresponding optimal values, found based on the ingredients in the inventory, are reflected in the top-right panel. The dashed lines represent each of the in-stock ingredients, and the circles indicate the suggested quantities to use by the system.

Given the presence of several viable solutions when optimising food processes, this real-world problem poses itself as a challenging task with an inherently underdetermined characteristic [2,3]. This work proposes an evolutionary computation technique as the mean to identify diverse solutions, each meeting quality constraints. We resort to a swarm intelligence algorithm, dispersive flies optimisation (DFO) [4], to optimise beer recipes based on predetermined organoleptic properties. Such an approach enables the use of an automated *quantitative ingredients selection*, which as of today, constitutes one of the primary experimental aspects of brewing. Figure 1 presents a high-level schematic view of the brewing process optimisation.

Our method rests on uncovering an appropriate mapping of brewing elements and target properties: such mapping is initially explored via the “reverse engineering” of some major labels produced with known properties, which also allows us to characterise consistent and validated recipes as a prerequisite to implement a generative approach. Learning these mappings provides a strong foundation and consistent internal principles for the generation of novel and alternative solutions. We then explore our systems’ ability to adapt to various on-the-fly changes to users preferences, affecting organoleptic properties, or ingredients’ consumption level (while staying as close as possible to the predefined organoleptic properties). Our motivation is to demonstrate our proposed method’s flexibility which can be seen as bringing benefits not just to the design phase but also potentially to the production phase where it can be used for added customisation, or mitigating unforeseen issues arising during the deployment of the method into production.

### Related Work

Beer brewing has attracted several attempts at optimising or automating various elements of the process. These have however, most often, considered specific or causal relationships between ingredients and isolated properties known to play a significant role in consumers’ preferences (e.g., foamability, flavour profile, temperature, aroma, and processing time). One such research by Ermi et al. [5] explores two deep learning architectures to model the nonlinear relationship between beer in these two domains with the aim of *classifying* coarse- and fine-grained beer types and *predicting ranges* for original gravity (OG), final gravity (FG), alcohol by volume (ABV), international bitterness units (IBU), and colour.

Viejo et al. [6] researched beer foamability where robotics and computer vision techniques were combined with noninvasive consumer biometrics to assess quality traits from beer foamability. It is known that foam-related parameters are associated with beer quality and dependent on the protein content. A recent study explores the development of a machine learning model to predict the pattern and presence of 54 proteins [7].

Furthermore, in another study, an objective predictive model is developed to investigate the intensity levels of sensory descriptors in beer using the physical measurements of colour and foam-related parameters, where a robotic pourer was used to obtain some colour and foam-related parameters from a number of different commercial beer samples [8]. It is claimed that this method could be useful as a rapid screening procedure to evaluate beer quality at the end of the production line. Developing techniques to assess quality traits and sensory analysis of beers through virtual simulations before the brewing process can play an important role in product development, lowering costs while maintaining specific quality traits and sensory profiles. Our own approach falls into this category, some of the grounding for our simulation being derived from reverse engineering of brand crafts. A recent work [9] investigates this by using sonication and traditional brewing techniques, with results demonstrating that the models developed using supervised machine learning based on near-infrared spectroscopy can accurately estimate physicochemical parameters.

Optimising the aroma profile has been another areas of interest. For instance, Trelea et al. [10] obtained various desired final aroma profiles while reducing the total processing time using the dynamic optimisation of three control variables: temperature, top pressure, and initial yeast concentration in the fermentation tank; the optimisation is based on a sequential quadratic programming algorithm on top of a dynamic model of alcoholic fermentation and on an aroma production model. Another recent work assesses the final aromatic profiles and physicochemical characteristics of beers [11]. This work presents artificial intelligence models based on aroma profiles, chemometrics, and chemical fingerprinting obtained from 20 commercial beers used as targets.

On a related topic, mash separation is known to be a critical preprocessing step in beer production where a high-quality stream of solubilised grain carbohydrates and nutrients is fed to the fermentors. Recent work by Shen et al. performed a sensitivity analysis towards mash separation improvements [12]. It concluded that strong wort volume and incoming feed quality to the mash filter have the strongest effect on filtration time, which it sees as a key performance metric for process optimisation.

In another beer optimisation-related task by Charry-Parra et al. [13], a technique was developed for the identification and quantification of volatile compounds of beer. This validation methodology enables its use as a quality control procedure for beer flavour analysis. A computational implementation of a kinetic model has also been proposed to rapidly generate temperature manipulations, simulating the operation of each candidate profile [14]. In related research by Rodman et al. [15], the aim has been to gain insight into the brewing process and provide an investigation into the influence of byproduct (diacetyl and ethyl acetate) threshold levels on obtainable fermentation performance by computing optimal operating temperature profiles for a range of constraint levels on byproduct concentrations in the final product. Some recent research has also been exploring faults detection in beers using artificial intelligence methods, as well as using strain development methodology to breed industrial brewing yeast [16,17].

In summary, previous studies have used various artificial intelligence techniques, sometimes combined with modelling approaches, targetting fermentation, foam, aroma profile, predicting beer flavours, and controlling of beer fermentation process. Despite the previous use of deep learning (DL) techniques, the majority of approaches can still be categorised as traditional optimisation models, some based on process (e.g., kinetic) modelling, and others on specific relationship between limited number of key variables. To some extent, even the previous use of DL was more focussed on establishing direct relationships between parameters than uncovering global behaviour.

To address the novel challenge of reverse brewing, our work proposes to use a gradient-free, evolutionary approach to facilitate the discovery of diverse, yet high fidelity and novel recipes, while taking into account both users’ preferences and their constraints. Our approach takes advantage of some unique properties of evolutionary approaches in terms of optimisation dynamics and the exploration of the solution space not directly covered by previous methods. We design a unified framework for optimisation without incorporating formulaic knowledge of some of the underlying processes (i.e. Process knowledge is only used for the calculation of optimisation fitness functions), taking advantage of the ability of evolutionary processes to perform both optimisation and process modelling, potentially improving solution coverage over previous work.

In this paper, Section 2 presents the system architecture, followed by an introduction to the evolutionary method underpinning the optimisation and solution generation. Subsequently, the experiments are described along with the overall methodology and performance measures to evaluate the system with real-world input. Section 3 reports on the experiments results and provides discussion on the algorithm’s performance when optimising more than twenty products with a diverse set of properties. Finally, in Section 4, the paper is concluded by presenting ongoing and future work.

## 2. Material and Methods

This section first proposes the system architecture in Section 2.1 and the evolutionary method in Section 2.2. Then, a set of experiments are presented in Section 2.3 where organoleptic properties of a selection of beers are used along with a real-world in-stock inventory to evaluate the proposed approach by “reverse manufacturing” these commercial beers from their target organoleptic properties which are obtained from publicly available data.

### 2.1. System Architecture

In the first instance, evolutionary modelling is able to accommodate a complex set of variables and optimisation parameters. Our population-based algorithm takes an inventory of existing ingredients and their weights (see Table 1) along with a desired set of organoleptic properties for a number of brands (see Table 2) and returns optimal ingredient lists and their associated amounts which facilitate the production of the target product.

**Figure 2:**
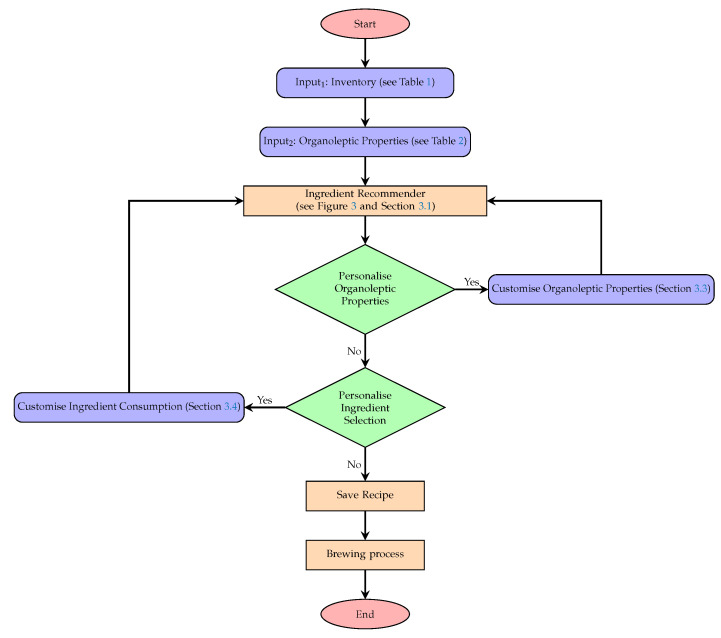
System architecture (please see Table 1 and Table 2, Figure 3, Section 3.1, Section 3.3 and Section 3.4).

Figure 1 provides a schematic view of the brewing process optimisation where the list of ingredients are provided on the left, along with their weights in the inventory. The organoleptic properties are provided by the user (in the top-left corner), reflecting their preferences. The system output, recommending the recipe of the best matching users’ preferences, is shown on the right along with their corresponding organoleptic properties. The underlying processes guiding the system are illustrated in Figure 2, along with pointers to various experiments in the paper which evaluates different proposed features.

The formulas which allow the simulation of the fermentation process are provided in Appendix A.1. As previously mentioned, this process modelling component is primarily used to determine fitness functions rather than to directly guide optimisation itself.

### 2.2. Population-Based Optimiser

The algorithm used in this work is a population-based optimiser, dispersive flies optimisation (DFO) [4], which unlike many other evolutionary algorithms, uses a minimalist set of vector/parameters [18,19]. In a preliminary study conducted recently [20], DFO has been used alongside other well-known population-based optimisers, including: particle swarm optimisation (PSO) [21], as one of the most well-known population-based algorithms, and differential evolution (DE) [22], a well-known and efficient evolutionary computation method. It has been demonstrated that DFO has outperformed these algorithms and is used as the optimiser in this work. This algorithm belongs to the broad family of swarm intelligence and evolutionary computation techniques and has been applied to a diverse set of problems including: medical imaging [23], solving diophantine equations [24], PID speed control of DC motor [25], optimising machine learning algorithms [26,27], training deep neural networks for false alarm detection in intensive care units [28], computer vision and quantifying symmetrical complexities [29], identifying animation key points from medialness maps [30], and the analysis of autopoiesis in computational creativity [31].

In this algorithm, the position vector (candidate solution (each solution is a vector whose length is equal to the number of existing ingredients, and each value in the vector represents the amount used from each ingredient.)) of each member of the population (the collection of candidate solutions) is defined as:

(1) $${\vec{x}}_{i}^{t}=\left[x_{i0}^{t},x_{i1}^{t},...,x_{i,D-1}^{t}\right],\;i\in \left\{0,1,2,...,N-1\right\}$$

where *i* represents the *i*’th individual (i.e., *i*’th solution), *t* is the current time step, *D* is the problem dimensionality (i.e., the number of ingredients), and *N* is the population size (i.e., the number of candidate solutions used for information exchange and communication, as is explained). For continuous problems, $x_{id}\in R$ (or a subset of R, which is the case of this problem, where $x_{id}$ is between 0 and the existing amount of the *d*’th ingredient).

In the first iteration, where t=0, the *d*’th component (or ingredient) of the *i*’th candidate solution is initialised as:

(2) $$x_{id}^{t=0}=U\left(x_{\min,d},x_{\max,d}\right)$$

where $x_{\min,d}=0$ and $x_{\max,d}$ is the maximum amount available to use for the *d*’th ingredient (see Table 1).

Components of the solution vectors are independently updated in each iteration, taking into account: current individual’s solution; current individual’s best neighbouring solution (consider ring topology, where individuals have left and right neighbours, each holding a solution vector); and the best solution vector in the swarm.

The update equation is

(3) $$\begin{array}{ccc} x_{id}^{t+1} & = & x_{i_{n}d}^{t}+u\left(x_{sd}^{t}-x_{id}^{t}\right) \end{array}$$

 where

- $x_{id}^{t}$: *d*’th ingredient of the *i*’th solution at time step *t*;
- $x_{i_{n}d}^{t}$: *d*’th ingredient of the solution vector held by ${\vec{x}}_{i}^{t}$’s best *neighbouring* individual (in ring topology) in at time step *t*;
- $x_{sd}^{t}$: *d*’th ingredient of the *swarm*’s best solution vector at time step *t*;
- $u∼U\left(0,1\right)$: generated afresh for each ingredient and for each solution update.

The optimisation process avoids local minima through a sampling-based restart mechanism. As a sampling mechanism, the individual ingredients’ amounts of the solution vectors are reset if a random number generated from a uniform distribution on the unit interval $U\left(0,1\right)$ is less than the disturbance or *restart threshold*, Δ=0.001.

**Figure 3:**
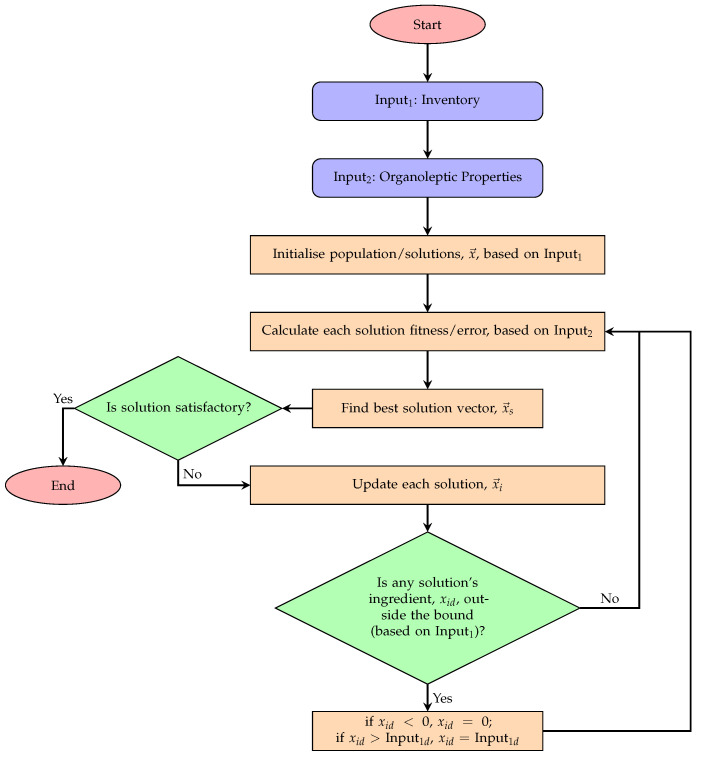
Ingredient recommender.

Elitism is used for DFO, which essentially keeps the best found solution in each iteration intact, while updating other solutions in the population. In this work, if the updated amount of an ingredient is outside the feasible boundaries, its value is clamped to the edges (i.e., to either 0 or the maximum existing amount of that particular ingredient). Algorithm 1 provides an overview of the process in which the algorithm performs the optimisation task,  and Figure 3 illustrates the steps taken as part of the ingredient recommendation process. The algorithm’s source code is available on: http://github.com/mohmaj/DFO (accessed on 6 January 2022). 

**Algorithm 1:** Dispersive Flies Optimisation (DFO)

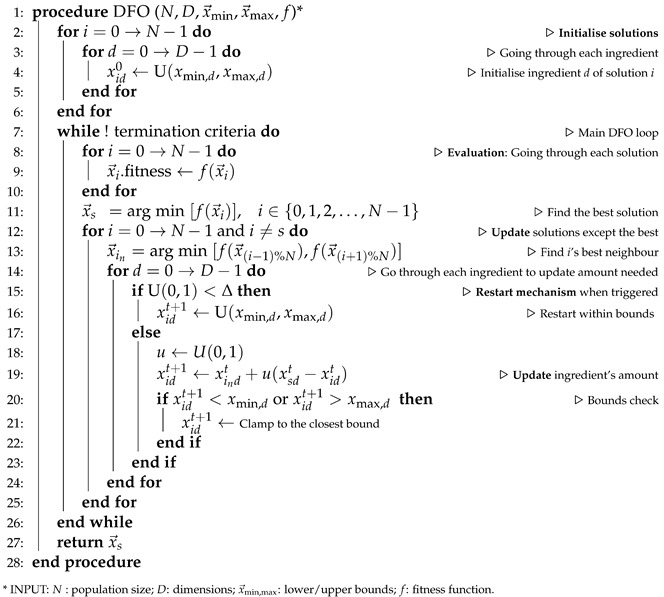



### 2.3. Experiment Setup

The first set of experiments gives an indication on the overall performance of the system on each product when generating solutions (whose diversity and distinctness are demonstrated in subsequent sections). This is then followed by analysing the behaviour of the algorithm in terms of improvements throughout the process. Then, the next set of experiments evaluates some practical features of the system, showing in particular its adaptability towards: (1) drastic, on-the-fly changes in organoleptic properties, (2) gradual changes in organoleptic properties during the optimisation process, and (3) system’s ability to accept real-time user-preference on the balance of ingredients’ consumption while preserving the user-defined organoleptic properties.

In order to set up the simulation experiments, we first adopted an inventory of ingredients, along with organoleptic properties of twenty-two existing commercial beers which were used as benchmarks. In these experiments, the population size for the DFO algorithm was set to 50, and the termination criterion was set to reaching 50,000 function evaluation or FEs (a counter which is incremented when a solution vector’s quality is evaluated) or reaching the error ≤0.05, which is defined next. There were 50 independent runs for each experiment, and the results are summarised over these independent simulations.

### 2.4. Performance Measures

The performance measures used in this paper are (a) error: representing the proximity to optimal solutions (this metric is used to steer the optimisation process); (b) efficiency: the speed of convergence to optimal solutions; (c) reliability: the consistency of the algorithm over a number of trials in reaching the optimal solutions; and (d) diversity: novelty of solutions and their uniqueness measured by their distance from each other.

*Error* is defined by the quality of the solution in terms of its closeness to the optimum position (i.e., minimisation).

(4) $$\begin{array}{c} \mathrm{Error}=f\left(\vec{x}\right)=\sum_{i=1}^{N_{p}}\sqrt{{\left(f_{p_{i}}\left(\vec{x}\right)-p_{i}^{*}\right)}^{2}} \end{array}$$

where $\vec{x}$ is the list of ingredients and $N_{p}=5$ is the number of properties, with $p_{1}$: ABV, $p_{2}$: IBU, $p_{3}$: Colour, $p_{4}$: OG, and $p_{5}$: FG (where the relevant equations are provided in Appendix A.1). $p_{i}^{*}$ represents the desired value provided by the brewers (in this case from Table 2), whose distance is measured against the value of the solution generated by the system.

*Efficiency* is defined as the number of function evaluations before reaching a specified error, and *reliability* is the percentage of trials where the specified error of ≤0.05 is reached.

(5) $$\begin{array}{ccc} \mathrm{Efficiency} & = & \frac{1}{n}\sum_{i=1}^{n}\mathrm{FEs}, \end{array}$$

(6) $$\begin{array}{ccc} \mathrm{Reliability} & = & \frac{n^{{}^{\prime }}}{n}\times 100 \end{array}$$

where *n* is the number of trials in the experiment and $n^{{}^{\prime }}$ is the number of successful trials. Additionally, *diversity* is used to measure the ability to explore the solution space towards producing multiple solutions. There are various approaches to measure diversity. The average distance around the population centre is shown to be a robust measure in the presence of outliers [32]:

(7) $$\begin{array}{c} \mathrm{Diversity}=\frac{1}{N}\sum_{i=1}^{N}\sqrt{\sum_{d=1}^{D}{\left(x_{id}-{\bar{x}}_{d}\right)}^{2}},\;\;\;{\bar{x}}_{d}=\frac{1}{N}\sum_{i=1}^{N}x_{id} \end{array}$$

where *N* is the population size and ${\bar{x}}_{d}$ is the average value of ingredient (or dimension) *d* over all solutions in the population. The experiment setup for this proof of principle work is based on simulating a realistic small-scale brewery, where the brewer’s efficiency is set to 58% (this refers to the efficiency of equipment in extracting sugars from malts during the mashing stage; efficiency is higher for larger-scale industrial setups), boil size of 24 L, batch size of 20 L, and boil time are set to 60 min.

## 3. Results and Discussion

**Figure 4:**
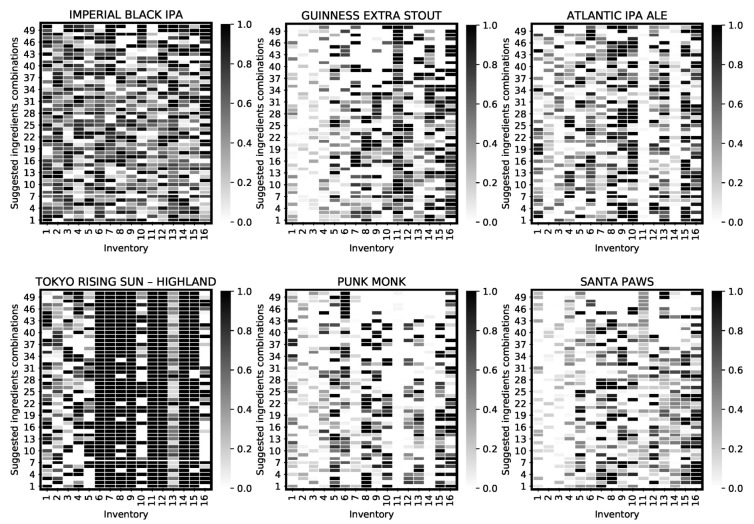
Ingredients combinations generated by the optimising system for the first six products. This illustrates recommended ingredients uptake proportion, as well as independent solutions’ diversity for each of the products. The complete set of ingredients combinations is provided in Appendix A.2 in Figure A1.

To demonstrate the process, Figure 4 illustrates 50 normalised solution vectors for the first six products generated by the system. These vectors visualise various viable ingredients combinations and the uptake of each of the input ingredients when reaching the termination point. In addition to the evident solution diversity illustrated in the visualisations, we later discuss the presence of some visible inclination (e.g., product 4) towards specific ingredients.

### 3.1. Accuracy, Efficiency, Reliability, and Diversity

Table 3 presents the results which demonstrate the system’s ability in finding solutions for 77% of the products to the required level of accuracy (error ≤0.05). The remaining 23% of the products (i.e., products 4, 7, 14, 17, and 20) share a common trait for which no acceptable solutions can be found considering the overall constraints on ingredients’ use; this common characteristic is the stronger colour or the SRM >70 (see Table 2). Achieving these strong colours requires more quantities of certain ingredients such as roasted barley which allows for the desired colours to be acquired. This limitation by ingredients quantities and distribution can however be circumvented by the optimisation algorithm by suggesting potentially acceptable substitutions still resulting in the satisfaction of target properties. The system illustrates its approach to compromise for the lack of sufficient ingredients in the inventory by trying others which could assist with, in this case, colour strength; looking at the fourth product and the level of fermentable consumption in Figure 4 (the product in the first column and the second row), it can be observed that 8 out of 10 existing fermentables (see Table 1) are used in order to approach the desired colour.

In other words, in more than three quarter of the products, where the required ingredients are available, the system finds an optimal solution. In addition, there are several indications of the consistency of system performance. One such indicator is derived from the internal behaviour of the evolutionary algorithm and can be expressed in terms of population diversity values for both successful and failed trials (see Table 3). This measure illustrates the presence of diversity irrespective of finding the solutions.

Furthermore, Table 4 reports on the efficiency measure which is the speed of the optimiser at reaching an optimal solution (i.e., the number of function evaluations, FEs, as shown in Equation (5)). To reach the optimal solution, the median FEs range between 950 and 5675, with the median of overall efficiency over the entire set of optimised products being 1150 FEs (out of the allowed 50,000 FEs). In terms of reliability, which measures the frequency of `performing optimally’ (and excluding the “impossible” cases), the optimiser exhibits a reliability above 84% for all the products (see Table 4).

### 3.2. Solution Vectors Diversity

**Figure 5:**
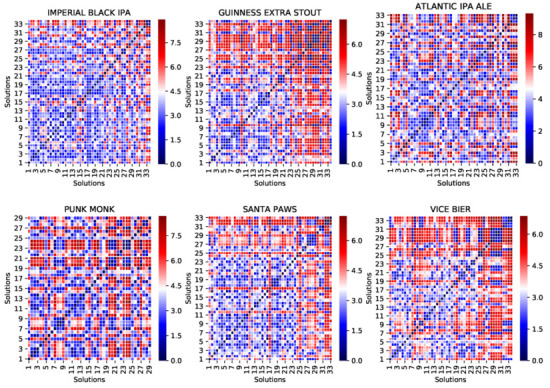
Solution vector distances. Visualising the distance matrices of the solution vectors in the first six optimised products (i.e., products 1, 2, 3, 5, 6, and 8). Note that, over the entire set of products, products 4, 7, and 14 have not been optimised. As for the rest of the products, only the valid solutions are considered (e.g., while Imperial Black IPA has 50 valid solutions, Punk Monk has 43). Table 5 presents the numerical summary of solution distance matrices, and the complete set of matrices is provided in Appendix A.2 in Figure A2.

Another objective we assigned to our approach, besides providing near-optimal solutions, is to diversify the set of potential solutions as a way to support more personalised or inventive formulations. To evaluate the uniqueness of the solution vectors, the distances between each pair of solutions (generated independently for each product) are studied. These values are presented as distance matrices in Figure 5.

One of the practical implications of having `distant’ solutions is their potentially different ingredient-combinations. In other words, in some extreme cases, some ingredients might be consumed entirely in one solution while remaining untouched in another, making `unique’ solutions/recipes available to users, thereby allowing them to choose based on their target properties and processing constraints. A practical illustration of the latter phenomenon is provided towards the end of this section.

To numerically analyse the solution diversity, Table 5 demonstrates that the products’ average solution diversity ranges consistently between 2.964 and 4.713. The distances among solutions can be further scrutinised by looking at the distance range in the most farther apart pairs when optimising the ingredients for each product.

Given the presence of diversity in the solution vectors, an essential task is to identify the most diverse solutions in a way that can support exploration and choice. This can be achieved by clustering solutions in a distinct set of `classes’ based on their propinquity. This process would grant users the freedom to choose distinct solutions by picking a solution from each independent cluster. Once the unique solutions are identified, users choose the most suitable based on their production priorities. To identify distinct clusters when utilising K-means [33] and to find the best number of clusters for each of the products (and avoid relying solely on one method), twenty indices [34] (e.g., [35,36,37,38,39,40,41]) are used; then, the ‘majority rule’ is applied to find the best number of clusters.

**Figure 6:**
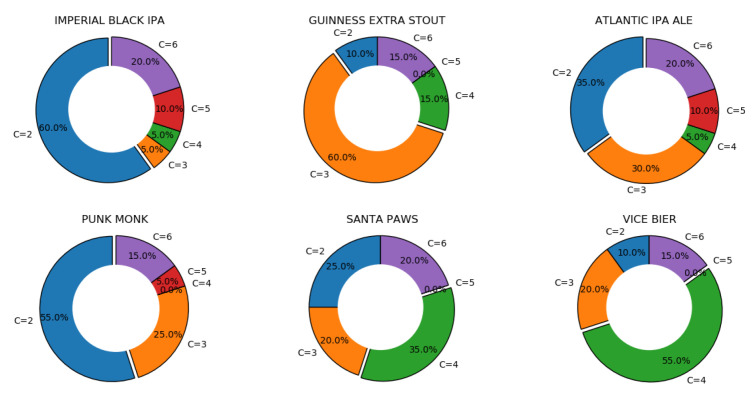
Strength of the possible number of clusters (based on the indices) in six products, where C={2,...,6} represents the number of clusters. The strength of each proposed number of clusters is determined by taking into account 20 clustering indices. The complete set of charts are provided in Appendix A.2 in Figure A3.

Figure 6 and Table 6 demonstrates that 65% of the products have at least 3 or more clusters of solutions. In 20% of the products, the best number of clusters is 6 (which is used as the upper bound in the calculations). Furthermore, except for one, 94% of the products have at least one index presenting six clusters as an optimum clustering strategy.

**Figure 7:**
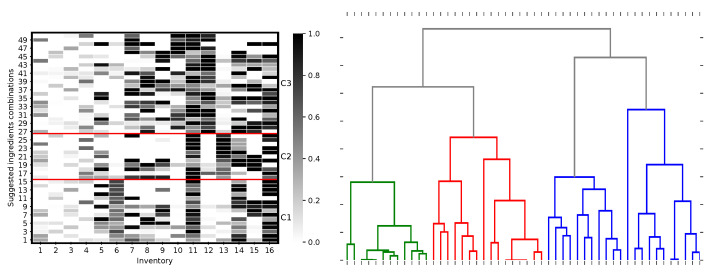
**Left**: Ordered solutions for Guinness Extra Stout based on clusters, C${}_{1}$, C${}_{2}$, C${}_{3}$, with C${}_{1}$: 1–15, C${}_{2}$: 16–26, and C${}_{3}$: 27–50). **Right**: Dendrogram of the clusters where colours green, red, and blue represent the aforementioned clusters.

As a case study, the solution clusters for the second product (Guinness Extra Stout) is analysed, and as evident from Figure 7-left, what primarily distinguishes the three identified clusters is the consumption of the following ingredients: 6th, 10th, 12th, and 13th. Based on this, Figure 7-right illustrates the three classes through a dendrogram, and Table 7 provides the detail of clusters’ characteristics in terms of the cluster-dependent, ingredient consumption. This illustrates that, in addition to the uniqueness of individual solutions themselves, distance thresholds between clusters can be analysed further by using methods such as hierarchical clustering approaches, which would be a topic for further work.

### 3.3. Dynamic Organoleptic Changes: Drastic and Gradual Modes

The experiments in this section evaluate the system’s ability to adapt to rapid changes in user’s preferences without the need to restart the optimisation process. These changes are expressed in terms of the target organoleptic properties. In the initial experiment, the system’s behaviour is examined when target properties are updated from one product to the next during the optimisation process (e.g., consider three products, 1, 2, and 3; each is optimised in turn, and the optimiser selects the next one, in a loop). The aim of this experiment is to evaluate the system’s ability in handling user’s change of “options” from one product to the next during the optimisation process. To proceed, initially one set of the organoleptic properties, belonging to item 1 (e.g., Guinness Extra Stout) is selected, and the optimiser is run until the target error value is reached; then, another product, item 2 (Kozel Black) is selected, followed by item 3 (Imperial Black IPA). This is repeated, and the properties are set to item 1 again and so forth. This process is illustrated in one trial demonstrating the system’s performance in adapting to the user’s drastic changes from one product to the next.

**Figure 8:**
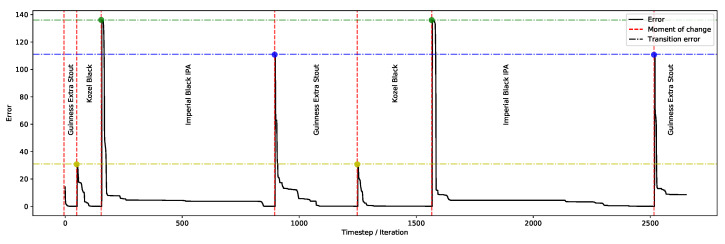
Applying drastic changes to preferences: switching from one product to the next. The dashed vertical lines represent the moment of change from one set of organoleptic properties to the next, and the horizontal lines present the transition error for each of the products.

Figure 8 illustrates the error values over the iterations and highlights the timing of change from one set of user preferences to another. The transition error, which is the transitioning from one set of organoleptic properties to the next, reflects the distance between the two sets. Another noticeable feature is the shorter time required when commencing the optimisation which `stretches’ over time. For instance, see item 1 which reaches an acceptable error value in nearly 50 iterations, whereas when transitioning from item 3 (where members of the population are mainly concentrated on this particular item), the personalisation takes nearly 350 iterations to readjust to the `newly’ chosen product. Despite the extended convergence time, the ability to operate in radically altered landscapes could be attributed to the algorithm’s persistent diversity (even after convergence) as discussed in Section 3.1.

While the possibility to switch from one product to the next is an essential feature, the ability to operate when minor changes are applied is also important in practice for various purposes, including the adaptation to marketing needs and fine-tuning adjustments to support, for instance, end-product localisation.

**Figure 9:**
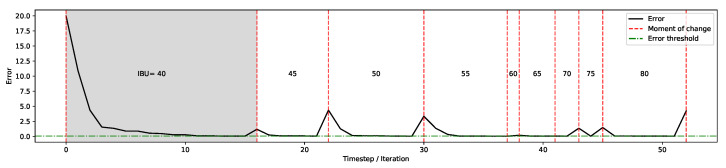
Applying gradual changes to preferences: gradually increasing IBU for Guinness Extra Stout by 5. The figure shows the gradual change (from IBU =40 onwards) requires less than half the time steps to accommodate user’s updated preferences.

To investigate the system’s performance in dealing with these gradual changes, in another run, the IBU value of Guinness Extra Stout is incrementally increased by 5, from 40 to 80. The performance of the system in this setup is illustrated in Figure 9, where it is shown that the error value “bounces back” and the system identifies new sets of ingredients to match the updated IBU. Note that other than the start of the process when the optimiser is converging to the solution (the shaded area), in the remaining part, the population self-organises faster around the new desired features. Achieving the original user preference (i.e., the organoleptic properties for Guinness Extra Stout) takes 16 iterations; however, from this point onward, the gradual changes to the user’s IBU preference only require on average 4.5 iterations. This illustrates the ability of our specific evolutionary algorithm to adapt to real-time changes in optimisation parameters. As a consequence, our approach can cope with users’ on-the-fly gradual adjustments of properties, thereby allowing a smooth fine-tuning of parameters, without the need to restart the entire optimisation process.

### 3.4. User-Defined Ingredient Consumption

From a practical perspective, another important feature required by small-scale brewers and industry brewers alike is the ability to consume a user-defined supply of certain ingredients while accommodating the required organoleptic properties. The rationale is the presence of certain ‘favourite’ ingredients whose quantities brewers may wish to fix. This can be because of their flavours, marketing preferences, ingredient costs, or other physicochemical characteristics, thereby exercising autonomy of choice rather than reliance on the optimiser. As outlined in the system architecture, when loading the inventory, users can specify the amounts they would like considered as part of the recipe discovery process. For the purpose of the experiment here and to evaluate the impact on the optimisation process, the usage of certain ingredients is set to correspond to their full consumption. Therefore, users can select the ingredients they would like consumed completely for their current brew ‘on-the-fly’ while observing (in ‘real-time’) the impact on discovering the right recipe with the desired organoleptic properties.

**Figure 10:**
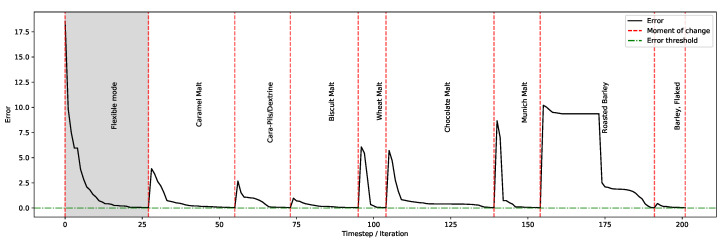
User selection of one ingredient at a time for full consumption.

To test this feature and as illustrated in Figure 10, initially, the system finds a recipe for Guinness Extra Stout using the existing inventory, and then each of the fermentables (see Table 1) are singled out in turn and set to be consumed entirely (except for the heaviest ingredients: ‘Pale Malt’ and ‘Pilsner’, which are 7 and 5 kg, respectively); this process continues until a recipe is discovered. Subsequently, the existing constraint is retired, and then the next fermentable is set to be individually consumed and so forth. The results illustrate the system’s ability to dynamically update recipes to reflect users’ needs and preferences in varying conditions.

In summary, we have shown the potential of the swarm intelligence method in the optimisation of various aspects of the brewing process. Since this first proof of concept was based on simulated data, we demonstrated a mechanism for the “reverse brewing” of commercial brands by taking into account the desired organoleptic properties and an inventory of ingredients in order to validate the algorithm with real-world data. In future work, when further objectives are considered, the use of multiobjective optimisation techniques (and by extension, multicriteria decision making methods) becomes necessary in order to investigate the Pareto front and subsequently identify the nondominated, Pareto optimal solutions [42,43,44]. In this work, in addition to using the presented system as an analytical tool to discover ideal and distinctive recipes, the reported experiments demonstrate adaptability and flexibility in dealing with a number of users preferences.

## 4. Conclusions

In this work, a population-based evolutionary technique is used to automate the quantitative *ingredients selection*, which is one of the key experimental aspects of brewing, specially in low- and medium-cost production environments. The performance of the presented tool was evaluated on a diverse set of real-world products to ensure its generalised applicability in generating solutions with distinctive features.

This work analyses the system’s adaptability on a number of practical and experimental scenarios with ‘on-the-fly’ changes in users preferences. The system’s ability in dealing with the properties of one product to the next was investigated, as well as its behaviour in adapting to gradual changes in some of the organoleptic properties. Additionally, the optimiser’s capability in exploring user preferences for consuming certain ingredients (while adhering to the predefined organoleptic properties) was examined.

In summary, the proposed approach alleviates the challenges of producing novel recipes based on their organoleptic properties. This is an attractive feature for both commercial producers where varieties and quantities of ingredients are not hard constraints and, in less equipped setups such as microbrewing, with stronger ingredients-based constraints, allowing the design of high-quality beer. Our results suggest that evolutionary methods should find their place in the panel of optimisation techniques: they are able to incorporate process modelling through their fitness functions, without being constrained or determined by a detailed modelling of the process itself, which would compromise solution exploration and interactive solution tuning; they can be interfaced with standard representations for ingredients selection; and most importantly, they can reconcile optimisation with the exploration of solution diversity.

Considering the high experimental cost associated with innovation in the beer brewing process, the approach we have introduced constitutes an optimiser and an analytical tool, which could be used from a simulation perspective or as a way to guide real-time experiments.

As part of the future work, we will add the more complex flavour and aroma profiles as well as foam characteristics, which are dependent, among others, on the fermentables and hops.

## Figures and Tables

**Table 1:** Real-world list of in-stock inventory.

	Type	Name	Amount
1	Hop	Cascade	100 g
2		Chinook	100 g
3		Northern Brewer	100 g
4		Magnum	40 g
5		Fuggles	50 g
6	Fermentable	Pale Malt (UK)	7 kg
7		Caramel/Crystal Malt	1 kg
8		Cara-Pils/Dextrine	1 kg
9		Biscuit Malt	0.5 kg
10		Wheat Malt (Belgium)	2 kg
11		Chocolate Malt (UK)	0.5 kg
12		Munich Malt	3 kg
13		Pilsner (German)	5 kg
14		Roasted Barley	0.5 kg
15		Barley Flaked	0.5 kg
16	Yeast	Safale S-04	11 mL

**Table 2:** Beer characteristics in a diverse set of products.

No.	Product Name	ABV	IBU	SRM	OG	FG
1	Imperial Black IPA	12.2	150	35	1.098	1.013
2	Guinness Extra Stout	5.1	40	40	1.070	1.034
3	Atlantic IPA Ale	8.4	80	13	1.074	1.013
4	Tokyo Rising Sun	15.4	85	71	1.125	1.023
5	Punk Monk	6.2	60	8.5	1.056	1.010
6	Santa Paws	4.7	35	22	1.048	1.013
7	Sunmaid Stout	11.1	50	100	1.102	1.026
8	Vice Bier	4.4	25	15	1.043	1.010
9	Blitz Berliner Weisse	4.3	8	4.5	1.040	1.007
10	Jasmine IPA	6.3	40	17.5	1.060	1.014
11	No Label	4.5	25	5	1.043	1.009
12	Monk Hammer	7.5	250	7.5	1.065	1.010
13	Science IPA	5.2	45	47	1.050	1.011
14	Tropic Thunder	7.5	25	86.36	1.074	1.020
15	Blonde Export Stout	7.7	55	8	1.075	1.020
16	Indie Pale Ale	4.8	30	8	1.044	1.008
17	Paradox Islay	14.2	40	127	1.112	1.015
18	Funk X Punk	7.2	42	12	1.058	1.004
19	Atlantic IPA Ale	8.4	80	28	1.074	1.013
20	Libertine Porter	6.5	45	109.5	1.067	1.020
21	Kozel Dark	4.6	35.09	21.87	1.042	1.007
22	Punk IPA	5.6	40	7.6	1.053	1.011
	Range:	[4.3,15.4]	[8,250]	[4.5,127]	[1.040,1.125]	[1.004,1.034]

**Table 3:** Performance results in error and diversity.

Error (Equation (4))						Diversity (Equation (7))	
Product No.	Best	Worst	Median	Mean	StDev	Successful	Failed
1	0.0202	0.0499	0.0406	0.0394	0.0083	0.8401	–
2	0.0301	0.0500	0.0435	0.0420	0.0055	0.9113	–
3	0.0115	1.1399	0.0398	0.0602	0.1561	0.9421	0.4770
4	12.6852	12.7703	12.6852	12.6908	0.0175	–	0.5770
5	0.0147	0.4489	0.0449	0.0543	0.0638	1.0140	0.1350
6	0.0094	0.0539	0.0386	0.0361	0.0119	1.0760	0.0296
7	41.9895	41.9923	41.9895	41.9896	0.0004	–	0.7550
8	0.0051	0.0494	0.0360	0.0352	0.0091	1.1087	–
9	0.0188	0.0500	0.0394	0.0368	0.0094	0.9120	–
10	0.0126	0.1253	0.0406	0.0439	0.0260	1.0135	0.5090
11	0.0112	0.1976	0.0402	0.0414	0.0262	0.8911	0.0295
12	0.0175	1.1881	0.0475	0.1240	0.2348	0.7943	0.3480
13	0.0077	0.0495	0.0368	0.0362	0.0090	0.8648	–
14	28.9527	28.9527	28.9527	28.9527	0	–	0.7050
15	0.0175	0.1936	0.0408	0.0474	0.0344	0.9778	0.4130
16	0.0178	1.4823	0.0403	0.0770	0.2100	0.9306	0.4740
17	68.4574	68.4574	68.4574	68.4574	0	–	0.7390
18	0.0229	0.0499	0.0409	0.0396	0.0073	1.3104	–
19	0.0147	0.0500	0.0382	0.0370	0.0085	0.789	–
20	51.9948	51.9948	51.9948	51.9948	0	–	1.3700
21	0.0086	0.1128	0.0406	0.0389	0.0144	1.0997	0.0553
22	0.0075	0.0496	0.0364	0.0361	0.0095	0.8024	–

**Table 4:** Optimisation performance: efficiency and reliability.

Efficiency (Equation (5))				Reliability (Equation (6))	
Product No.	Best	Worst	Median	Cases	Percentage
1	600	18,050	1150	50	100
2	700	35,150	1150	50	100
3	600	–	1100	49	98
4	–	–	–	0	0
5	550	–	1250	43	86
6	700	–	950	49	98
7	–	–	–	0	0
8	500	30,300	1075	50	100
9	550	37,600	4125	50	100
10	600	–	1050	46	92
11	550	–	5675	47	94
12	650	–	3050	42	84
13	600	36,500	1000	50	100
14	–	–	–	0	0
15	600	–	1550	45	90
16	600	–	2400	47	94
17	–	–	–	0	0
18	700	8300	975	50	100
19	750	2200	1050	50	100
20	–	–	–	0	0
21	650	39,550	1150	49	98
22	600	49,100	2200	50	100

**Table 5:** Solutions diversity: statistics.

Product No.	Mean	Min Distance	Max Distance	StDev	Farthest Pair
1	3.910	0.494	8.921	1.527	(47, 49)
2	3.887	0.033	7.226	1.630	(37, 42)
3	4.554	0.354	9.302	1.992	(3, 46)
5	4.376	0.026	8.666	2.030	(24, 30)
6	3.546	0.071	7.213	1.402	(47, 48)
8	3.700	0.044	6.852	1.478	(38, 45)
9	2.964	0.012	5.727	1.638	(4, 17)
10	3.992	0.028	8.693	1.568	(38, 44)
11	3.289	0.018	6.451	1.613	(36, 45)
12	4.139	0.117	8.855	2.093	(0, 40)
13	3.652	0.010	7.230	1.486	(47, 48)
15	4.713	0.032	8.885	2.636	(33, 43)
16	3.946	0.037	7.376	1.757	(31, 44)
18	4.678	0.222	8.879	2.189	(47, 48)
19	4.123	0.592	8.778	1.732	(33, 37)
21	3.987	0.007	7.094	1.705	(43, 48)
22	4.437	0.023	8.207	2.249	(21, 49)

**Table 6:** Solution clusters. The figures present the total number of solutions in each cluster when the best clustering strategy (using majority rule) is used.

Product	Clust 1	Clust 2	Clust 3	Clust 4	Clust 5	Clust 6	Majority
Imperial Black IPA	22 (44%)	28 (56%)	–	–	–	–	12 (60%)
Guinness Extra Stout	15 (30%)	11 (22%)	24 (48%)	–	–	–	12 (60%)
Atlantic IPA Ale	26 (53%)	23 (47%)	–	–	–	–	7 (35%)
Punk Monk	17 (40%)	26 (60%)	–	–	–	–	11 (55%)
Santa Paws	15 (30%)	15 (30%)	9 (18%)	11 (22%)	–	–	7 (35%)
Vice Bier	10 (20%)	12 (24%)	14 (28%)	14 (28%)	–	–	11 (55%)
Blitz Berliner Weisse	16 (32%)	3 (6%)	13 (26%)	18 (36%)	–	–	10 (50%)
Jasmine IPA	8 (17%)	11 (24%)	9 (20%)	8 (17%)	6 (13%)	4 (9%)	8 (40%)
No Label	16 (34%)	13 (28%)	18 (58%)	–	–	–	15 (75%)
Monk Hammer	27 (64%)	15 (36%)		–	–	–	12 (60%)
Science IPA	7 (14%)	14 (28%)	5 (10%)	5 (10%)	7 (14%)	12 (24%)	9 (45%)
Blonde Export Stout	26 (58%)	19 (42%)	–	–	–	–	12 (60%)
Indie Pale Ale	23 (49%)	11 (23%)	13 (28%)	–	–	–	11 (55%)
Funk X Punk	16 (32%)	16 (32%)	18 (36%)	–	–	–	7 (35%)
Atlantic IPA Ale	27 (54%)	23 (46%)	–	–	–	–	12 (60%)
Kozel Dark	16 (33%)	16 (33%)	17 (35%)	–	–	–	7 (35%)
Punk IPA	7 (14%)	14 (28%)	19 (38%)	10 (20%)	–	–	7 (35%)

**Table 7:** Clusters’ distinct characteristics in Guinness Extra Stout, illustrating varying ingredient consumptions.

	Pale Malt	Wheat Malt	Munich Malt	Pilsner
Cluster 1	HIGH			
Cluster 2				HIGH
Cluster 3		HIGH	HIGH	

**Figure A1:**
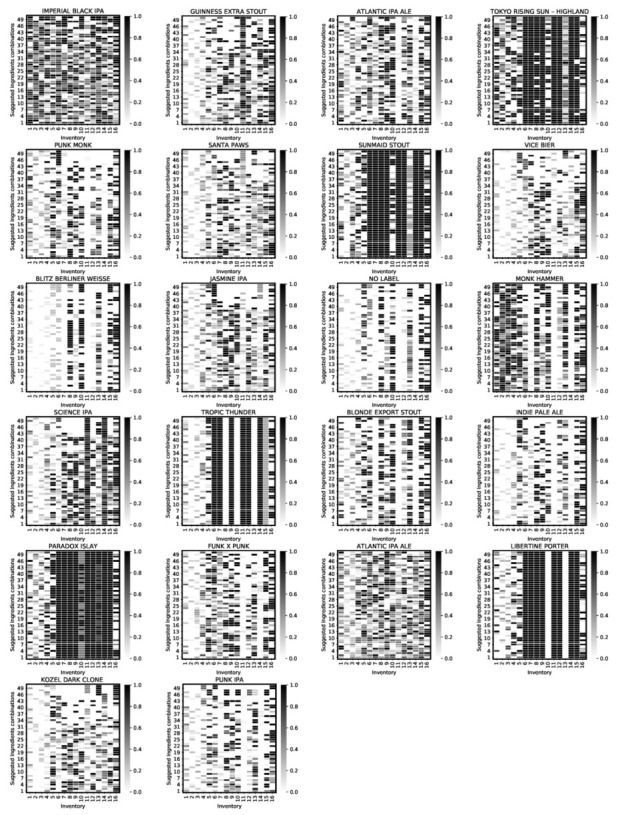
Ingredients combinations generated by the system. This illustrates the recommended ingredients uptake proportion and the independent solutions’ diversity for each product.

**Figure A2:**
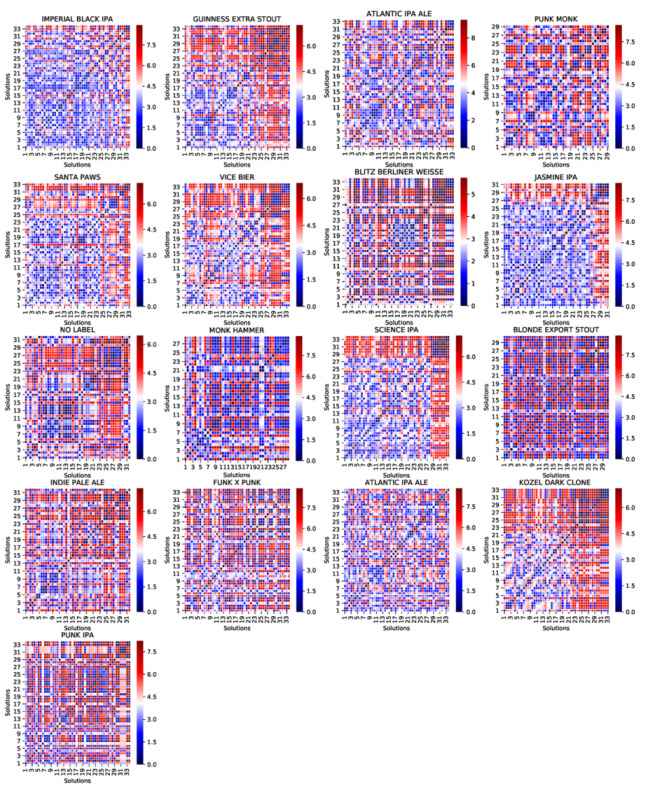
Solution vector distances. Visualising the distance matrices of the solution vector in the first 12 products. Note that products 4, 7, and 14 are not included as they have not been optimised. As for the rest of the products, only the valid solutions are considered (e.g., while Imperial Black IPA has 50 valid solutions, Punk Monk has 43). These graphs illustrate the diversity in the generated solutions. Table 5 presents the numerical summary of solution distance matrices.

**Figure A3:**
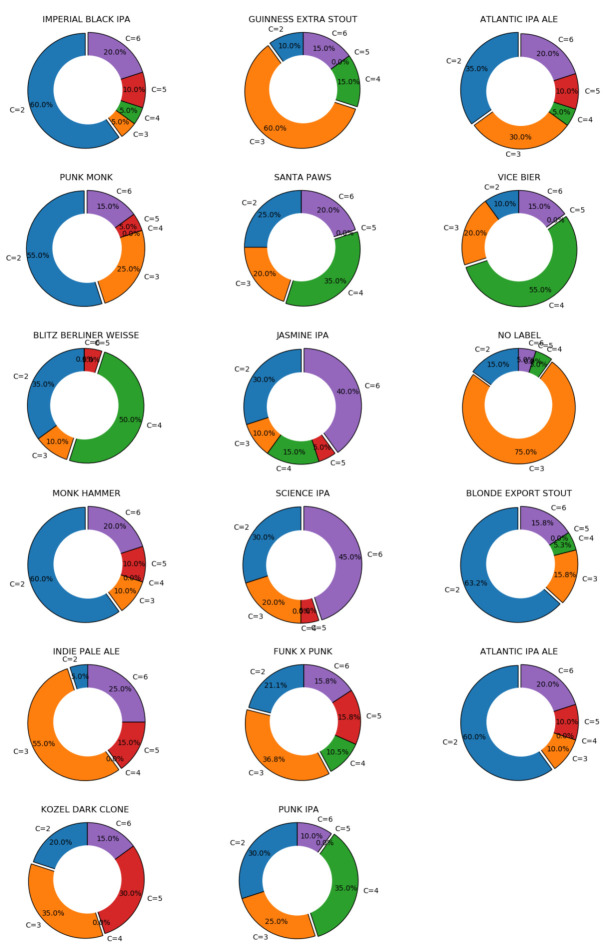
Strength of the possible number of clusters in each product, where C={2,...,6} represents the number of clusters. The strength of each proposed number of clusters is determined by taking into account 20 clustering indices.

## Data Availability

Not applicable.

## Appendix A. Process Equations, List of Visualised Solutions, Distances and Clusters

### Appendix A.1. Process Equations and Optimisation Variables

In the brewing process, ingredients are divided in three broad categories: hops, fermentables, or yeasts. In addition to weight, several other relevant physicochemical features are needed to calculate their impact in the brewing process (e.g., hop’s alpha and beta; fermentable’s yield, colour, moisture and diastatic power; yeast’s minimum and maximum temperatures; and attenuation). Beer’s taste changes significantly depending on the exact quantities and varieties of ingredients and their timing in the process. The key organoleptic properties which contribute towards computing the fitness value of the solutions are alcohol by volume (ABV), bitterness or international bitterness unit (IBU), and colour, which are used by the optimiser to determine the suitability of each proposed solution. From a food science perspective, the brewing process, although in some parts empirical, has been the subject of many descriptions and partial formalisation, which are, however, sufficient to derive relevant equations. More specifically, a number of formal relationships between ingredients and target organoleptic properties are sufficiently specific to support the generation of fitness functions. Some of the relevant formulas are discussed next.

**ABV**=f(OG,FG) and is defined as [45]:

(A1) $$\begin{array}{c} \mathrm{ABV}=131.25\times (\mathrm{OG}-\mathrm{FG}) \end{array}$$

When ABV is above 6% or 7%, the following is used, providing a higher level of accuracy [46,47]:

(A2) $$\begin{array}{c} \mathrm{ABV}=\frac{76.08\;(\mathrm{OG}-\mathrm{FG})\mathrm{FG}}{0.794\;(1.775-\mathrm{OG})} \end{array}$$

**IBU** is determined by taking into account the bitterness produced by hops or the hop extracts (from the fermentables), thus $\mathrm{IBU}=f(\vec{\mathrm{hops}},\vec{\mathrm{fermentables}},\mathrm{volume})$. The bitterness produced by hops is calculated as follows:

(A3) $$\begin{array}{c} {\mathrm{IBU}}_{h}=\sum_{i=1}^{N_{h}}\frac{10w_{i}\alpha_{i}\left(1-\exp^{-0.04t_{i}}\right)}{4.15\;v}1.65\times 0.000125^{\left(\mathrm{OG}-1\right)} \end{array}$$

where $N_{h}$ is the number of hops; $\vec{w}$ represents the weight; *v* is the volume or batch size; $\vec{t}$ is time in minutes; and fermentables’ bitterness is defined as:

(A4) $$\begin{array}{c} {\mathrm{IBU}}_{f}=\sum_{i=1}^{N_{f}}\frac{g_{i}\;w_{i}}{v} \end{array}$$

where $N_{f}$ is the number of fermentables; and $\vec{g}$ is ‘IBU gal per lb’ which is associated with each fermentable and is known for each ingredients. The final IBU is the sum of the individual IBUs: $\mathrm{IBU}={\mathrm{IBU}}_{h}+{\mathrm{IBU}}_{f}$.

**IBU/GU** is often described in the following categories: cloying, slightly malty, balanced, slightly hoppy, extra hoppy, and very hoppy. IBU/GU=f(OG,IBU):

(A5) $$\begin{array}{c} \mathrm{IBU}/\mathrm{GU}=\frac{\mathrm{IBU}}{1000(\mathrm{OG}-1)} \end{array}$$

**Colour** is mainly determined by malts and hops. The two main protocols to measure colour are Standard Reference Method (SRM) and European Brewing Convention (EBC). Table A1 shows the representative colours. SRM, which is used in this work, was initially adopted in 1950 by the American Society of Brewing Chemists. The value of SRM is determined by measuring the attenuation of light of a particular wavelength (430 nm) in passing through 1 cm of the beer, expressing the attenuation as an absorption and scaling the absorption by a constant (12.7 for SRM or 25 for EBC, where EBC=SRM×1.97). Stone and Miller [48] proposed malt colour unit (MCU), which is defined as:

(A6) $$\begin{array}{c} \mathrm{MCU}=\sum_{i=1}^{N_{f}}\frac{c_{i}\;w_{i}}{v} \end{array}$$

where $\vec{c}$ refers to grains’ colour (fermentables’ colour). As shown in the equation above, for more than one grain type, the MUC is calculated for each, and all the values are aggregated. However, MUC tends to overestimate the colour value for darker beers (MUC >10.5). Thus, Morey [49] derived the following to deal with SRM up to 50:

(A7) $$\begin{array}{c} \mathrm{SRM}=1.4922\sum_{i=1}^{N_{f}}\frac{c_{i}\;w_{i}^{0.6859}}{v} \end{array}$$

**Table A1:** Beer colour in SRM and EBC values.

	SRM	EBC	Colour
	2	4	Pale Straw
	3	6	Straw
	4	8	Pale Gold
	6	12	Deep Gold
	9	18	Pale Amber
	12	24	Medium Amber
	15	30	Deep Amber
	18	35	Amber-Brown
	20	39	Brown
	24	47	Ruby Brown
	30	59	Deep Brown
	40+	79	Black
